# Superbunching and Nonclassicality as new Hallmarks of Superradiance

**DOI:** 10.1038/srep17335

**Published:** 2015-12-03

**Authors:** Daniel Bhatti, Joachim von Zanthier, Girish S. Agarwal

**Affiliations:** 1 Institut für Optik, Information und Photonik, Universität Erlangen-Nürnberg, 91058 Erlangen, Germany; 2 Department of Physics, Oklahoma State University, Stillwater, OK, USA; 3 Erlangen Graduate School in Advanced Optical Technologies (SAOT), Universität Erlangen-Nürnberg, 91052 Erlangen, Germany

## Abstract

Superradiance, i.e., spontaneous emission of coherent radiation by an ensemble of two-level atoms in collective states introduced by Dicke in 1954, is one of the enigmatic problems of quantum optics. The startling gist is that even though the atoms have no dipole moment they radiate with increased intensity in particular directions. Following the advances in our understanding of superradiant emission by atoms in entangled *W*-states we examine the quantum statistical properties of superradiance. Such investigations require the system to have at least two excitations in order to explore the photon-photon correlations of the radiation emitted by such states. We present specifically results for the spatially resolved photon-photon correlations of systems prepared in doubly excited *W*-states and give conditions when the atomic system emits nonclassial light. Equally, we derive the conditions for the occurrence of bunching and even of superbunching, a rare phenomenon otherwise known only from nonclassical states of light like the squeezed vacuum. We finally investigate the photon-photon cross correlations of the spontaneously scattered light and highlight the nonclassicalty of such correlations. The theoretical findings can be implemented with current technology, e.g., using ions in a linear rf-trap, atoms in an optical lattice or quantum dots in a cavity.

Dicke^1^ predicted that if an ensemble of two-level atoms is prepared in a collective state where half of the atoms are in the excited state and half of the atoms are in the ground state the spontaneous emission is proportional to the square of the number of atoms as if the particles would radiate coherently in phase like synchronized antennas^2^. To analyze the phenomenon Dicke introduced the concept of collective spins where *N* two level atoms are described by the collective spin eigenstates 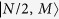, with *M* running from 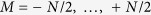 in steps of unity. Among these states the state 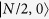 radiates with an intensity 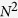 times as strong as that of a single atom. The origin of superradiance is difficult to see since all states 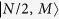 exhibit no macroscopic dipole moment whereas such a dipole moment is commonly assumed to be required for a radiation rate proportional to 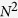. The reason is that the Dicke states display strong quantum entanglement. The entangled character of the states is particularly apparent for the case of two two-level atoms where the individual atomic states are labeled by 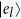 and 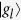, 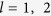, for the excited and ground state, respectively. In this case the Dicke state 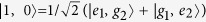, also known as the Bell state or the EPR state, is clearly maximally entangled. For three atoms one of the Dicke states is denoted by |3/2,  −1/2, which in current language would be the *W*-state 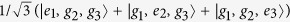
3. The single excited generalized *W*-state, where only one atom is excited and 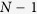 atoms are in the ground state, is also known to be fully entangled and plays a particularly important role for single-photon superradiance^4^,^5^,^6^,^7^,^8^. In fact, it has been recognized that most of the important aspects of superradiance^1^,^9^,^10^,^11^,^12^ can be studied by examining samples in single excited generalized *W*-states^4^,^5^,^7^,^8^,^13^,^14^,^15^,^16^,^17^,^18^ as the emission from these states possesses all the features of superradiance that originally were calculated for samples with an arbitrary number of excitations^5^,^15^. The spatial features of one-photon superradiance have been extensively studied for example from the perspective of *timed Dicke states*^4^,^5^,^8^ and also the spectral and temporal aspects have been investigated in a large variety of systems^7^,^14^,^18^,^19^,^20^,^21^,^22^. Note that a number of recent works^23^,^24^,^25^,^26^,^27^,^28^,^29^ have also discussed how single excited generalized *W*-states for a small number of atoms can be prepared in the laboratory.

The single excited generalized *W*-state does however not allow one to study the quantum statistical properties of superradiance. In order to explore these aspects the system must emit at least two photons. Only then one has access to the photon-photon correlations which display amongst others the particular quantum characteristics of the spontaneously scattered radiation^30^,^31^,^32^. To this end it is required to investigate what we will term two-photon superradiance from generalized *W*-states with 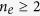 excitations.

In the present paper we show that in two-photon superradiance the emitted radiation can exhibit both bunched as well as nonclassical and antibunched light depending on the angle of observation, i.e., the position of the detectors collecting the scattered photons, and on the particular *W*-state, i.e., the number of atoms *N* and the number of excitations 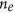, considered. In particular, in certain cases it is also possible to observe the phenomenon of superbunching, i.e., photon-photon correlations larger than those maximally measurable for classical light sources. In all the cases the mean intensity displays the familiar features of superradiance produced by the corresponding *W*-state. While we derive our results for two-photon superradiance for arbitrary generalized *W*-states we focus in this paper on systems in doubly excited *W*-states; the outcomes for arbitrary *W*-states with more than two excitations are presented in the Supplementary Information section.

Note that bunching in the radiation of generalized *W*-states can be explained semi-classically. However, the phenomenon of superbunching as well as the emission of nonclassical light, first demonstrated in 1977^31^, can only be understood in a quantum mechanical description^31^,^33^. The latter is a feature arising from the light’s particle nature where photon fluctuations become smaller than for coherent light. Demonstrating nonclassicality in the light of arbitrary *W*-states thus directly leads to a manifestation of the particular quantum mechanical characteristics of these superradiant states. We finally discuss also the spatial cross correlations of photons in two-photon superradiance. Here, likewise, superbunching and nonclassicality can be observed.

We note that recent investigations for a mesoscopic number of atoms in a cavity have already reported the observation of superbunching^34^,^35^. In this paper we bring out for a simple model system in free space the reasons for the appearance of this phenomenon, a curio which does not commonly occur, the squeezed vacuum being one of the rare examples^36^. The theoretical predictions could be verified experimentally, e.g., using ions localized in a linear rf-trap or atoms trapped in an optical lattice.

## Results

**Figure 1:**
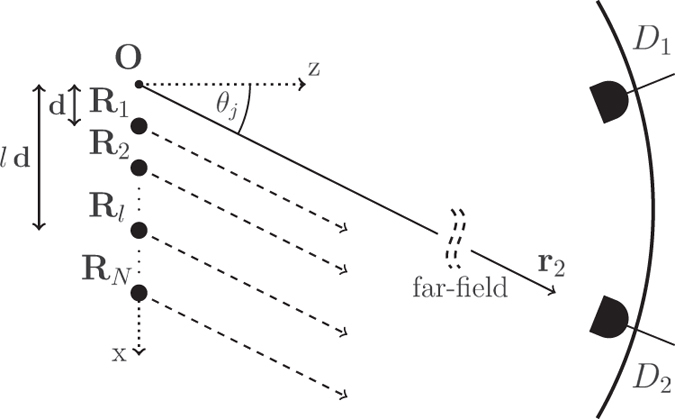
Scheme of considered setup: *N* two-level atoms are aligned on the x-axis, whereby neighboring atoms are separated by a distance *d*. The intensity in the far field 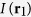 is measured by a single detector at position 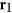, whereas the second order correlation function 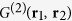 is measured by two detectors at 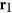 and 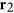.

To focus on the key aspects of two-photon superradiance we consider a linear system of *N* equidistantly aligned identical emitters, e.g., atoms or ions with upper state 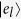 and ground state 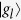, 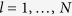, trapped in a linear arrangement^37^,^38^,^39^,^40^ at positions 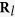 with spacing 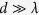 such that the dipole-dipole coupling between the particles can be neglected (see Fig. 1). This configuration - without any loss of generality - simplifies the calculations and leads to intuitive results which can be easily compared to an array of unentangled atoms emitting coherently in phase like a regular collection of synchronized antennae. The atoms are assumed to be prepared initially in a generalized *W*-state with 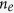 excitations, i.e., in the state 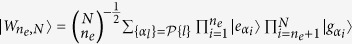, where 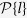 denotes all permutations of the set of atoms 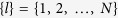. In what follows we study the second order correlation functions at equal times of the light emitted by the atoms in the described *W*-state. To this end two detectors are placed at positions 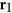 and 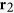 in the far field each measuring a single photon coincidentally, i.e., within a small time window much smaller than the lifetime of the upper state. To simplify the calculations we suppose that the emitters and the detectors are in one plane and that the atomic dipole moments of the transition 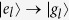 are oriented perpendicular to this plane (see Fig. 1).

Due to the far field condition and therefore the inability to identify the individual photon sources, the electric field operator at 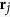 takes the form^41^ 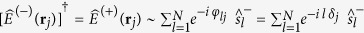, where 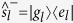 is the atomic lowering operator for atom 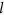, and 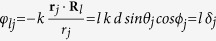 the relative optical phase accumulated by a photon emitted by source 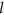 and recorded by detector *j* with respect to a photon emitted at the origin. Hereby, 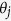 and 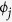 denote the polar angle and the azimuth angle of the 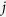-th detector, respectively. Note that the field operators have been chosen dimensionless as all dimension defining prefactors cancel out in the normalized correlation functions. The first and second order spatial correlation functions at equal times are defined as^30^ 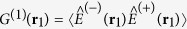 and 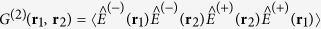, respectively, where 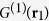 is proportional to the mean intensity of the emitted radiation, i.e., 
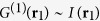
. To compare the photon statistics of various systems radiating with different intensities we further introduce the normalized second order correlation function^30^ 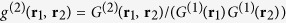.

For the state 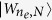 the first order correlation function in the configuration of Fig. 1 has been calculated^15^ to


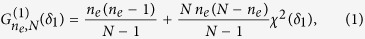


where 
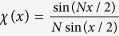
 corresponds to the normalized far-field intensity distribution of a coherently illuminated 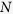-slit grating^42^. As it is well-known from classical optics, the distribution 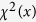 is strongly peaked in particular directions. The fact that 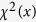 appears in the context of spontaneous emission of atoms in generalized *W*-states as in Eq. (1) has been coined by Dicke *spontaneous emission of coherent radiation* or simply *superradiance*^1^. Note that the function 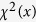 appears in Eq. (1) due to the symmetry of the setup displayed in Fig. 1. Note that even though 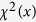 displays pronounced maxima in certain directions the distribution assumes also very small values and even vanishes in other directions what has been interpreted as *subradiance* of the states 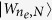
15.

From Eq. (1) we find that the intensity distribution of the state 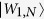 with only one excitation (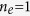) simplifies to 
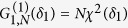
, with 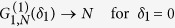, displaying a maximal visibility 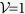 and a peak value *N* times the intensity of a single atom. This kind of single photon superradiance has been extensively studied in the past^4^,^5^,^7^,^8^,^14^,^15^,^16^,^17^,^18^. Its particular superradiant characteristics have been shown to result from quantum path interferences occurring due to the particular interatomic correlations of the collective state 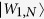
15.

As discussed below the strong correlations of the states 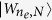 may lead to photon-photon correlations with 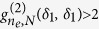 as well as 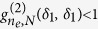, corresponding to superbunched as well as nonclassical light, respectively. Note that from the form of normalized second order correlation function 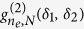 it is obvious that bunching necessitates small intensities, i.e., 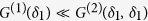, whereas nonclassical light requires small values of the two-photon correlation function, i.e., 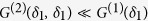.

In what follows we study two-photon superradiance for the simplest form of generalized *W*-states, i.e., *W*-states with only two excitations, as the main features of two-photon superradiance can already be observed for this configuration; two-photon superradiance for arbitrary generalized *W*-states 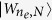 with 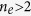 is discussed in the Supplementary Information section.

Note that an atomic ensemble in the state 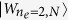 can be prepared for example by using photon pairs generated in a down conversion process. When sent on a 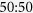 beam splitter the photon pair would then produce two photons at either of the two output ports of the beam splitter, i.e., if one port delivers zero photons then the other one has two photons^43^. Assuming perfect detection efficiency, a photon pair not registered at one output port of the beam splitter and not registered at the other one after having passed the atomic ensemble would then herald the absorption of two photons by the atomic system.

For 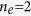 the normalized second order correlation function for the considered configuration of Fig. 1 takes the form


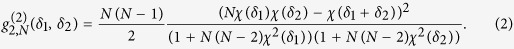


This expression will be investigated in detail in the following subsections.

### Superbunching in Two-photon Superradiance

In this section we investigate whether bunching in two-photon superradiance, in particular the phenomenon of superbunching with 
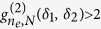
, can be observed in the radiation produced by states of the form 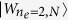. We start to explore the photon-photon correlations with the two detectors placed at equal positions, i.e., with the two spontaneously emitted photons recorded in the same mode; photon-photon cross correlations are studied thereafter.

According to Eq. (2) the second order correlation function for the state 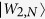 with two detectors placed at the same position takes the form


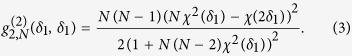


Note that this function is even with respect to 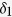 if adding an angle 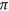 due to the symmetry of the setup (see Fig. 1).

**Figure 2:**
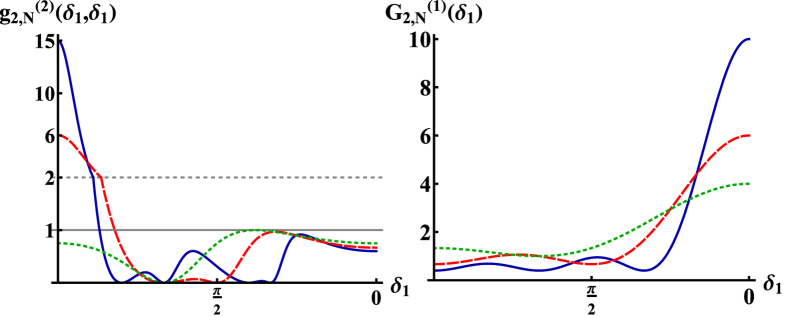
Second order correlation function 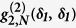 (left) and first order correlation function, i.e., the intensity distribution, 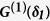 (right) of the radiation emitted by *N* two-level atoms in the doubly excited *W*-state 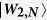 for *N* = 3 (dotted), *N* = 4 (dashed), *N* = 6 (solid). Superbunching is observed at 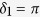 for 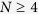, while antibunching occurs at detector positions 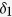 fulfilling 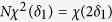. For increasing 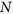 superbunching becomes stronger, similar to single photon superradiance of the state 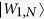. Comparing the two plots one can see that for high values of 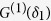 small values of 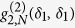 are obtained and *vice versa*.

In order to access whether the system displays bunching for this configuration we have to search for values 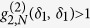. Hitherto, we choose detector positions for which the values of the first order correlation function 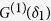 remain smaller than the unnormalized second order correlation function 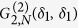, i.e., locations where the intensity is low. This occurs for example at 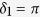, where 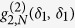 attains its maximal value (see Fig. 2). To investigate this outcome quantitatively we have to study the case of even and odd *N* separately since 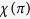 and 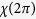 yield different results in these two cases. Note that in the regime 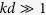 of widely spaced atoms we are investigating, it is simple to obtain 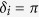 or 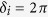, 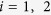, as these values can be achieved already with observation angles 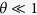 (see Fig. 1).

For an even number *N* of atoms one obtains the following two identities 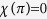, 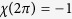, from which we deduce 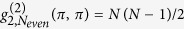, leading to bunched light in case that 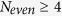 (see Fig. 2). In fact, for 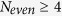, we even obtain superbunching, i.e., 
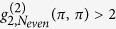
, what surpasses the maximum value achievable with classical light. Note that 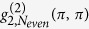 as a function of 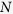 has in principal no upper limit as it increases 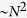 for 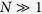. This means that we can produce principally unlimited values of superbunching if we add more and more atoms in the ground state to the system^34^.

In case of odd *N* the above identities read 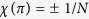, 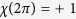, what leads to the maximal value of the second oder correlation function of 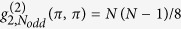. Here we obtain bunched and superbunched light in case that 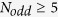. Again, as in the case of even *N*, there is no upper limit for the maximum value of the two-photon correlation function, as once more we have 
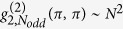
. The width of 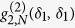 can be estimated by looking at the value of 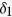 for which 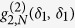 falls from its maximal value (at 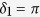) to 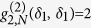. From Eq. (3) we obtain a scaling of the angular width 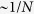. Hence, in order to observe the phenomenon of superbunching for large atomic ensembles 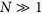, the angular resolution of the detector should be at least better than 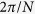.

### Nonclassicality in Two-photon Superradiance

In this section we investigate whether for the initial state 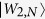 and two detectors at equal positions we can obtain nonclassical light, a sub-Poissonian photon statistics and antibunching in two-photon superradiance. As known from the radiation of a single atom^31^,^33^ a sub-Poissonian photon statistics derives from the discrete nature of the scattered radiation and can be explained only in a quantum mechanical treatment of resonance fluorescence. A complete vanishing of the second order correlation function at equal times for the state 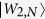 indicates that 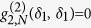 at 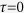 what proves true antibunching, whereas values 
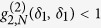
 result from the nonclassical nature of the radiation scattered by the state 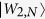.

Noting that the second order correlation function 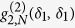 displays a visibility of 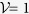 (see Fig. 2) there must be indeed detector positions where 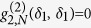. More specifically, according to Eq. (3), the photon-photon correlation function vanishes independently of the atom number *N* in case that the detectors are located at positions 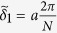, with 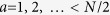, as in this case 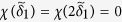. Towards these positions the atomic system thus radiates photons which display complete antibunching. More generally, for all detector positions 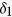 fulfilling 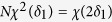 complete antibunching is obtained (cf. Eq. (3)). If the two detectors are located at 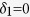 we have 
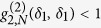
 as long as 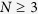, as in this case the photon-photon correlation function takes the form 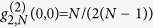 (cf. Eq. (3)). Hence, in those directions the atomic system emits nonclassical light with photon number fluctuations smaller than those for coherent light.

### Two-photon Cross Correlations

Finally we study the spatial cross correlations in two-photon superradiance for atoms in the state 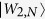, i.e, the behavior of the second order correlation function 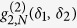 in case that the scattered photons are recorded at different positions 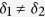. We start to explore the particular configuration of counter-propagating detectors, i.e., detectors at positions 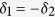, followed by the case where 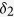 is fixed and only 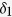 is varied.

For 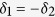 (i.e., where we choose 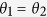, 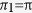 and 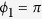), the second order correlation function reads (cf. Eq. (2))


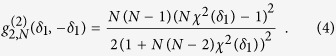


**Figure 3:**
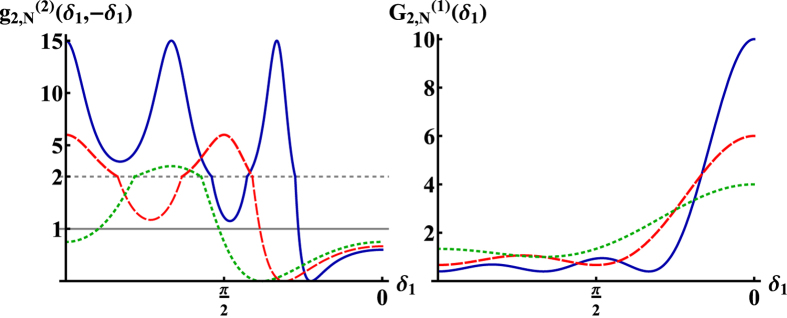
Left: Second order correlation function 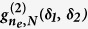 for 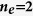, 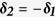 and *N* = 3 (dotted), *N* = 4 (dashed), *N* = 6 (solid). The function displays both superbunching and antibunching with a maximal visibilities of 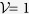. It can be seen that the superbunching effect increases with increasing *N*, while for 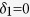 the second order correlation function converges to 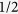. Right: First order correlation function 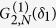 for *N* = 3 (dotted), *N* = 4 (dashed), *N* = 6 (solid). From the figure it can be seen that for small values of 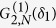 high values of 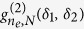 are obtained. In contrast to the case 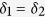 several superbunching peaks occur.

To determine the possibilities for superbunching in cross correlations of the scattered photons we look for the maximum of Eq. (4). This is attained at 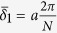, with 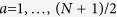, in which case 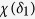 vanishes and the second order correlation function reads 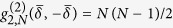. This result is principally identical to 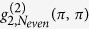. However, in the case of counter-propagating detectors it is valid for arbitrary 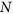, i.e., for 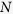 even or odd. Here, the threshold for superbunching is exceeded if 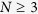 (see Fig. 3). Similarly as above for co-propagating photons, we estimate the width of 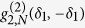 for counter-propagating photons by deriving the value of 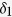 for which 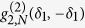 reduces from its maximal value at 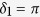 to the first local minimum. From Eq. (4) we obtain again a scaling of the angular width 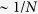. Hence, in order to observe the phenomenon of superbunching for large atomic ensembles *N* 1 in this case, the angular resolution of the detector should be better than 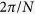.

In the same configuration also complete antibunching can be obtained. This occurs for 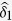 such that 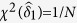 (cf. Eq. (4)), in which case the photon correlation function vanishes identically, i.e., 
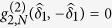
.

**Figure 4:**
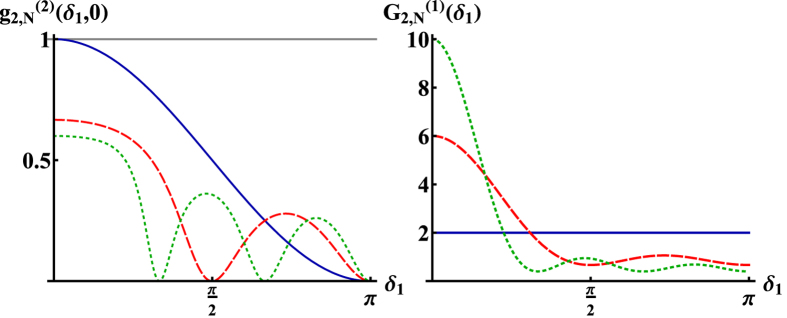
Left: Second order correlation function 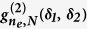 for 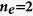 where one detector is fixed at 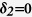. For all 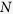 (in the plot: 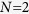 (solid), 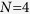 (dashed), 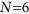 (dotted)) the two-photon correlation function remains smaller than one for any 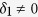. For 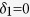 the maximal value of one is attained only in the case 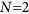. Right: First order correlation function 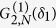 for 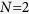 (solid), 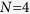 (dashed), 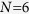 (dotted).

Another interesting configuration is the case when 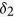 is fixed and only 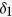 is varied. If we fix 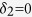 the photon-photon cross correlation function takes the form (cf. Eq. (2))


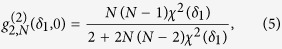


which can maximally take a value of 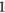 (for *N* = 2 and 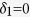), whereas for 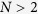 the cross correlation function remains always 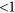. In Fig 4 the corresponding photon-photon cross correlations are shown for 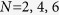 for the entire range 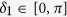. It can be seen that by increasing 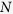 the second order correlation function 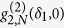 decreases in absolute values. The reason is the following: since 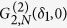 and 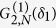 depend on 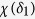 in a similar way the overall behavior of 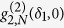 is determined by the prefactors of 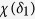 in the numerator and denominator of 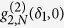, the ratio of which decreases with increasing 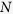 and converges to 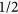 for 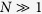.

## Discussion

In conclusion we investigated for a prototype ensemble of *N* identical non-interacting two-level atoms prepared in collective superradiant generalized *W*-states with 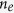 excitations the particular quantum statistical properties of the emitted radiation. Such investigations require the collective system to have at least two excitations as we explore the photon-photon correlations of the scattered light. We derived conditions for which the atomic system emits bunched and even superbunched light, as well as nonclassial and antibunched radiation. Here, superbunching refers to values of the normalized second order correlation function 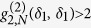 and antibunching to values 
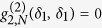
. In some cases the results were obtained under the condition that the number of atoms in the ensemble exceeds a certain threshold. For example, the smallest number of atoms producing superbunching in the state 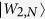 is 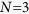; similar results were derived for 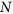 atoms with arbitrary number of excitations, as shown in the Supplementary Information section. Note that in coherently driven atomic systems superbunching can be observed already for *N* = 2. For example, in^44^ it was demonstrated that arbitrarily high values of 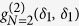 can be produced for 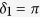 in case that the two atoms are very weakly excited, as in this case 
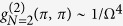
, where 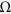 is the Rabi frequency (in units of the spontaneous decay rate 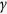). The effect is even stronger if the two atoms are subject to a strong dipole-dipole interaction as in dipole blockade systems; here 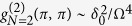 where 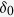 is the level shift of the doubly excited state (again in units of 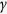)^45^. In the last part of the paper we finally investigated the spatial cross correlations in two-photon superradiance, i.e., the second order correlation functions 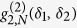 for detector positions 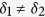. Here, again, positions were found where 
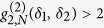
 and 
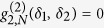
, corresponding in this case to superbunching and antibunching of the cross correlations of the scattered photons.

## Additional Information

**How to cite this article**: Bhatti, D. *et al.* Superbunching and Nonclassicality as new Hallmarks of Superradiance. *Sci. Rep.* **5**, 17335; doi: 10.1038/srep17335 (2015).

## Supplementary Material

Supplementary Information
