# Supplementary material for: Superbunching and Nonclassicality as new Hallmarks of Superradiance
Source: Sci Rep. 2015 Dec 3;5:17335. doi: 10.1038/srep17335 (PMC4668389; doi:10.1038/srep17335)
Supplement: Supplementary Information [file srep17335-s1.pdf]

# Supplementary Information Superbunching and Nonclassicality as new Hallmarks of Superradiance

**Daniel Bhatti[1,2,*], Joachim von Zanthier[3], and Girish S. Agarwal[2]**

[1]Institut für Optik, Information und Photonik, Universität Erlangen-Nürnberg, 91058 Erlangen, Germany
[2]Department of Physics, Oklahoma State University, Stillwater, OK, USA
[3]Erlangen Graduate School in Advanced Optical Technologies (SAOT), Universität Erlangen-Nürnberg, 91052 Erlangen, Germany
[*]daniel.bhatti@fau.de

## Photon-photon correlations for atoms in arbitrary generalized $W$-states

In this Supplementary Information section we calculate the second order correlation function for $N$ atoms in a generalized $W$-state $|W_{n_e,N}\rangle$, i.e., with an arbitrary number of excitations $n_e$. For this purpose the correlation function of second order can be written as

$$G^{(2)}_{n_e,N}(\delta_1,\delta_2) = \binom{N}{n_e}^{-1} \sum_{|\Psi_f\rangle} \Big| \sum_{l_1,l_2\in\{\Psi_f\}} e^{i(l_1\delta_1+l_2\delta_2)}\Big|^2 \,, \tag{S1}$$

with $|\Psi_f\rangle$ denoting all possible final states. Each final state is characterized by a particular set of $N-n_e+2$ integers $\{\Psi_f\}\subseteq\{1,\ldots,N\}$ representing all sources, that could have emitted the detected two photons. Since each source can emit only one photon, we have $l_1\neq l_2$. Note that for $n_e=2$ there is only one final state and the sum over $|\Psi_f\rangle$ vanishes. From Eq. (S1) one can find a combinatorial solution to the problem

$$G^{(2)}_{n_e,N}(\delta_1,\delta_2) = \binom{N}{n_e}^{-1} \sum_{\substack{l'_1,l'_2,l_1,l_2=1\\ l'_1\neq l'_2,\, l_1\neq l_2}}^{N-1} A_{l'_1,l'_2,l_1,l_2}\, e^{-i(l'_1\delta_1+l'_2\delta_2)}\, e^{i(l_1\delta_1+l_2\delta_2)} \,, \tag{S2}$$

where each term requires a statistical loading $A_{l'_1,l'_2,l_1,l_2}$ depending on the phase factors $l'_1\neq l'_2$ and $l_1\neq l_2$. With $m=(0\times2),(1\times2),(2\times2)$ of these factors being equal the statistical loading corresponds to

$$A_{l'_1,l'_2,l_1,l_2} = \binom{N-4+\frac{m}{2}}{n_e-2} \,, \tag{S3}$$

since there are $n_e-2$ excitations that can be distributed over $N-4+m/2$ atoms. This leads to (cf. Eq. (S2))

$$G^{(2)}_{n_e,N}(\delta_1,\delta_2) = \binom{N}{n_e}^{-1} \Big[ \binom{N-4}{n_e-2} B_{m=0} + \binom{N-3}{n_e-2} B_{m=2} + \binom{N-2}{n_e-2} B_{m=4} \Big] \,. \tag{S4}$$

We now calculate the different terms $B_m$ of $G^{(2)}_{n_e,N}(\delta_1,\delta_2)$ separately, starting with the case $m=4$

$$\begin{aligned} B_{m=4} &= \sum_{L\neq K} e^{-i(L\delta_1+K\delta_2)}\, e^{i(L\delta_1+K\delta_2)} + \sum_{L\neq K} e^{-i(L\delta_1+K\delta_2)}\, e^{i(K\delta_1+L\delta_2)} \\ &= \sum_{L,K=1}^{N} e^{-i(L\delta_1+K\delta_2)}\, e^{i(L\delta_1+K\delta_2)} - \sum_{L=1}^{N} e^{-iL(\delta_1+\delta_2)}\, e^{iL(\delta_1+\delta_2)} \\ &\quad + \sum_{L,K=1}^{N} e^{-i(L\delta_1+K\delta_2)}\, e^{i(K\delta_1+L\delta_2)} - \sum_{L=1}^{N} e^{-iL(\delta_1+\delta_2)}\, e^{iL(\delta_1+\delta_2)} \\ &= N^2-2N+N^2\chi^2(\delta_1-\delta_2) \,, \end{aligned} \tag{S5}$$

where in the last step the following identity has been used

$$\sum_{K,L=1}^{N} e^{iK\delta} e^{-iL\delta} = \frac{\sin^2(\frac{N\delta}{2})}{\sin^2(\frac{\delta}{2})} = N^2\chi^2(\delta)\ . \tag{S6}$$

$B_{m=4}$ (cf. Eq. (S5)) can now be used to calculate $B_{m=2}$

$$\begin{aligned}
B_{m=2} =& \sum_{L\neq l_2'\neq l_2\neq L} e^{-i(L\delta_1+l_2'\delta_2)}\, e^{i(L\delta_1+l_2\delta_2)} + \sum_{L\neq l_1'\neq l_1\neq L} e^{-i(l_1'\delta_1+L\delta_2)}\, e^{i(l_1\delta_1+L\delta_2)} \\
&+ \sum_{L\neq l_2'\neq l_1\neq L} e^{-i(L\delta_1+l_2'\delta_2)}\, e^{i(l_1\delta_1+L\delta_2)} + \sum_{L\neq l_1'\neq l_2\neq L} e^{-i(l_1'\delta_1+L\delta_2)}\, e^{i(L\delta_1+l_2\delta_2)} \\
=& \sum_{L\neq l_2', l_2} e^{-i(L\delta_1+l_2'\delta_2)}\, e^{i(L\delta_1+l_2\delta_2)} + \sum_{L\neq l_1', l_1} e^{-i(l_1'\delta_1+L\delta_2)}\, e^{i(l_1\delta_1+L\delta_2)} \\
&+ \sum_{L\neq l_2', l_1} e^{-i(L\delta_1+l_2'\delta_2)}\, e^{i(l_1\delta_1+L\delta_2)} + \sum_{L\neq l_1', l_2} e^{-i(l_1'\delta_1+L\delta_2)}\, e^{i(L\delta_1+l_2\delta_2)} - 2B_{m=4}\ .
\end{aligned} \tag{S7}$$

The two terms

$$\begin{aligned}
\sum_{L\neq l_2', l_2} e^{-i(L\delta_1+l_2'\delta_2)}\, e^{i(L\delta_1+l_2\delta_2)} =& \sum_{L,l_2',l_2=1}^{N} e^{-i(L\delta_1+l_2'\delta_2)}\, e^{i(L\delta_1+l_2\delta_2)} - \sum_{L,l_2'=1}^{N} e^{-i(L\delta_1+l_2'\delta_2)}\, e^{iL(\delta_1+\delta_2)} \\
&- \sum_{L,l_2=1}^{N} e^{-iL(\delta_1+\delta_2)}\, e^{i(L\delta_1+l_2\delta_2)} + \sum_{L=1}^{N} e^{-iL(\delta_1+\delta_2)}\, e^{iL(\delta_1+\delta_2)} \\
=& N + N^2(N-2)\chi^2(\delta_2)\ ,
\end{aligned} \tag{S8}$$

and

$$\begin{aligned}
\sum_{L\neq l_2', l_2} e^{-i(L\delta_1+l_2'\delta_2)}\, e^{i(l_1\delta_1+L\delta_2)} =& \sum_{L,l_2',l_2=1}^{N} e^{-i(L\delta_1+l_2'\delta_2)}\, e^{i(l_1\delta_1+L\delta_2)} - \sum_{L,l_1=1}^{N} e^{-iL(\delta_1+\delta_2)}\, e^{i(l_1\delta_1+L\delta_2)} \\
&- \sum_{L,l_2'=1}^{N} e^{-i(L\delta_1+l_2'\delta_2)}\, e^{iL(\delta_1+\delta_2)} + \sum_{L=1}^{N} e^{-iL(\delta_1+\delta_2)}\, e^{iL(\delta_1+\delta_2)} \\
=& N - N^2\chi^2(\delta_2) - N^2\chi^2(\delta_1) + \sum_{L,l_1,l_2=1}^{N} e^{iL(\delta_2-\delta_1)} e^{il_1\delta_1} e^{-il_2\delta_2}\ ,
\end{aligned} \tag{S9}$$

lead to the solution of Eq. (S7), which reads

$$B_{m=2} = -2N^2 + 8N + N^2(N-4)\,\chi^2(\delta_1) + N^2(N-4)\,\chi^2(\delta_2) - 2N^2\chi^2(\delta_1-\delta_2) + 2N^3\chi(\delta_1)\chi(\delta_2)\chi(\delta_1-\delta_2)\ , \tag{S10}$$

where the identity

$$\begin{aligned}
\sum_{L,l_1,l_2=1}^{N} e^{iL(\delta_2-\delta_1)} e^{il_1\delta_1} e^{-il_2\delta_2} + \sum_{L,l_1,l_2=1}^{N} e^{iL(\delta_1-\delta_2)} e^{il_1\delta_2} e^{-il_2\delta_1} &= 2\sum_{L,l_1,l_2=1}^{N} \cos(L(\delta_1-\delta_2)+l_1\delta_1-l_2\delta_2) \\
&= 2N^3\chi(\delta_1)\chi(\delta_2)\chi(\delta_1-\delta_2)\ ,
\end{aligned} \tag{S11}$$

has been used.

The last term $B_{m=0}$ of Eq. (S4) can now be calculated

$$\begin{aligned}
B_{m=0} =& \sum_{l_1'\neq l_2';\ l_1\neq l_2} e^{-i(l_1'\delta_1+l_2'\delta_2)}\, e^{i(l_1\delta_1+l_2\delta_2)} - B_{m=2} - B_{m=4} \\
=& \sum_{l_1',l_2',l_1,l_2=1}^{N} e^{-i(l_1'\delta_1+l_2'\delta_2)}\, e^{i(l_1\delta_1+l_2\delta_2)} - \sum_{l_1',l_2',L=1}^{N} e^{-i(l_1'\delta_1+l_2'\delta_2)}\, e^{iL(\delta_1+\delta_2)} \\
&- \sum_{L,l_1,l_2=1}^{N} e^{-iL(\delta_1+\delta_2)}\, e^{i(l_1\delta_1+l_2\delta_2)} + \sum_{L,K=1}^{N} e^{-iL(\delta_1+\delta_2)}\, e^{iK(\delta_1+\delta_2)} - B_{m=2} - B_{m=4} \\
=& N^2 - 6N - N^2(N-4)\,\chi^2(\delta_1) - N^2(N-4)\,\chi^2(\delta_2) + N^4\chi^2(\delta_1)\chi^2(\delta_2) + N^2\chi^2(\delta_1+\delta_2) + N^2\chi^2(\delta_1-\delta_2) \\
&- 2N^3\chi(\delta_1)\,\chi(\delta_2)\,\chi(\delta_1+\delta_2) - 2N^3\chi(\delta_1)\,\chi(\delta_2)\,\chi(\delta_1-\delta_2)\ ,
\end{aligned} \tag{S12}$$

where identities (S6) and (S11) have been used and $B_{m=2}$ (cf. Eq. (S10)) and $B_{m=4}$ (cf. Eq. (S5)) have been inserted.

Summing over all different terms the correlation function finally takes the form (cf. Eq. (S4))

$$\begin{aligned} G^{(2)}_{n_e,N}(\delta_1,\delta_2) = \binom{N}{n_e}^{-1} \Bigg[ \binom{N-4}{n_e-2} \Big[ & N^2-6N-N^2(N-4)\,\chi^2(\delta_1)-N^2(N-4)\,\chi^2(\delta_2) \\ & +N^4\chi^2(\delta_1)\chi^2(\delta_2)+N^2\chi^2(\delta_1+\delta_2)+N^2\chi^2(\delta_1-\delta_2) \\ & -2N^3\chi(\delta_1)\,\chi(\delta_2)\,\chi(\delta_1+\delta_2)-2N^3\chi(\delta_1)\,\chi(\delta_2)\,\chi(\delta_1-\delta_2)\Big] \\ +\binom{N-3}{n_e-2} \Big[ & -2N^2+8N+N^2(N-4)\,\chi^2(\delta_1)+N^2(N-4)\,\chi^2(\delta_2) \\ & -2N^2\chi^2(\delta_1-\delta_2)+2N^3\chi(\delta_1)\chi(\delta_2)\chi(\delta_1-\delta_2)\Big] \\ +\binom{N-2}{n_e-2} \Big[ & N^2-2N+N^2\chi^2(\delta_1-\delta_2)\Big] \Bigg] . \end{aligned} \tag{S13}$$

In the following two sections we investigate the second order correlation functions obtained in case of using generalized $W$-states $|W_{n_e,N}\rangle$ with higher numbers of excitations $n_e > 2$.

## Superbunching in the radiation of $N$ atoms in arbitrary generalized $W$-states

Similar to the case of $n_e = 2$ we want to investigate whether bunching and superbunching can be observed in two-photon superradiance from generalized $W$-states of higher excitations $|W_{n_e>2,N}\rangle$. We start to explore the photon-photon correlations with the two detectors placed at equal positions. In this case the normalized correlation function reads (cf. Eq. (S13))

$$\begin{aligned} g^{(2)}_{n_e,N}(\delta_1,\delta_1) = \mathscr{N} \binom{N}{ne}^{-1} \Bigg[ & \binom{N-2}{n_e-2} 2N\Big[N-1\Big] - \binom{N-3}{n_e-2} 4N\,(N-2)\Big[1-N\chi^2(\delta_1)\Big] \\ & + \binom{N-4}{n_e-2}\Big[2N(N-3)-4N^2(N-2)\,\chi^2(\delta_1)+\big(N\chi(2\delta_1)-N^2\chi^2(\delta_1)\big)^2\Big]\Bigg], \end{aligned} \tag{S14}$$

with the normalization $\mathscr{N} = (G^{(1)}_{n_e,N}(\delta_1))^{-2}$ (cf. Eq (1)).

To access whether the correlation function displays bunching we investigate the conditions for $g^{(2)}_{n_e,N} > 1$. As in the case $n_e = 2$, we choose the position $\delta_1 = \pi$ where $g^{(2)}_{n_e,N}$ attains its maximal value and the normalizing intensity is small (see Fig. S1). Since $\chi(\pi)$ and $\chi(2\pi)$ yield different results for even $N$ and odd $N$ these two cases have to be investigated separately.

In case of an even $N$ the second-order correlation function reads (cf. Eq. (S14))

$$g^{(2)}_{n_e,N_{even}}(\pi,\pi) = \frac{(N-1)(6+N^2+N-9n_e-2Nn_e+3n_e^2)}{(N-3)n_e(n_e-1)} . \tag{S15}$$

For large $N \gg n_e$ we can, as in case of $n_e = 2$, produce principally unlimited values of superbunching since there is no upper limit to $g^{(2)}_{n_e,N_{even}}(\pi,\pi)$ when adding more and more atoms in the ground state.[1] In this limit the correlation function simplifies to

$$g^{(2)}_{n_e,N_{even}}(\pi,\pi) \sim \frac{N^2}{n_e(n_e-1)}, \tag{S16}$$

clearly exceeding both the threshold for bunching ($g^{(2)}_{n_e,N} = 1$) as well as the threshold for superbunching ($g^{(2)}_{n_e,N} = 2$), whereby the fastest growth is obtained for $n_e = 2$. For example, for $n_e = 3$, Eq. (S15) is given by

$$g^{(2)}_{3,N_{even}}(\pi,\pi) = \frac{(N-1)(N-2)}{6} , \tag{S17}$$

leading to bunched and superbunched light for $N_{even} \geq 6$.

In case of odd $N$, the following maximal value of the second order correlation function can be calculated (cf. Eq. (S14))

$$g^{(2)}_{n_e,N_{odd}}(\pi,\pi) = \frac{N(n_e-1)(N^2+N-2Nn_e-5n_e+3n_e^2)}{n_e^3(N-2)} . \tag{S18}$$

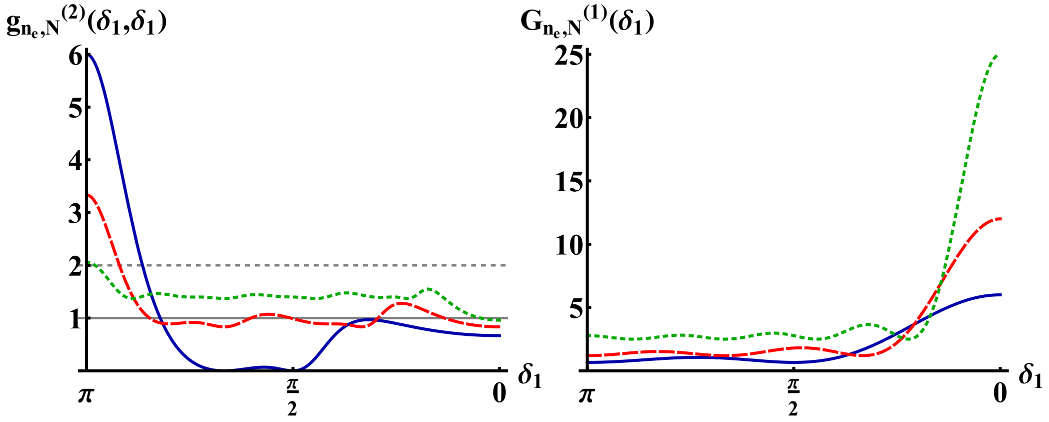


**Figure S1.** Second order correlation function $g^{(2)}_{n_e,N}(\delta_1,\delta_1)$ (left) and first order correlation function $G^{(1)}_{n_e,N}(\delta_1)$ (right) for $(n_e,N)=(2,4)$ (solid), $(3,6)$ (dashed), $(5,9)$ (dotted). For $\delta_1=\pi$ superbunching is observed for all displayed cases, whereas antibunching is obtained only for $n_e=2$, independently of $N$ (see Eq. (S14)). For $\delta_1=0$ the second order correlation function displays nonclassicality in case that $2(n_e-1)<N$ (see Eq. (S21)). Comparing the two plots one can see that for high values of $G^{(1)}_{n_e,N}(\delta_1)$ small values of $g^{(2)}_{n_e,N}(\delta_1,\delta_1)$ are obtained and vice versa.

From Eq. (S18) the behavior of the second order correlation function for $N\gg n_e$ can be derived

$$g^{(2)}_{n_e,N_{odd}}(\pi,\pi)\sim\frac{N^2(n_e-1)}{n_e^3}\,, \tag{S19}$$

which again may display bunching as well as superbunching, as there is no upper limit to Eq. (S19) when increasing $N$. For example, for $n_e=3$ and odd $N$ the second order correlation function reads

$$g^{(2)}_{3,N_{odd}}(\pi,\pi)=\frac{2N(N^2-5N+12)}{27(N-2)}\,, \tag{S20}$$

which shows bunching for $N_{odd}\geq 3$ and superbunching for $N_{odd}\geq 7$.

## Nonclassicality and antibunching in the radiation of $N$ atoms in arbitrary generalized $W$-states

We next investigate whether for initial arbitrary generalized $W$-states we can observe also nonclassical light and antibunching in two-photon superradiance. Again, we start to explore the photon-photon correlations with the two detectors placed at equal positions.

Note that a visibility of $\mathscr{V}=1$ of the second order correlation function is obtained only for initially doubly excited states, what rules out true antibunching for $n_e\geq 3$ (see Eq. (S14)). However, for increasing $n_e$, we can always find $g^{(2)}_{n_e,N}(\delta_1,\delta_1)<1$ at $\delta_1=0$, what can be explained physically since here the intensity attains its maximum $G^{(1)}_{n_e,N}(0)=n_e(N-n_e+1)$.[2] To prove this we calculate from Eq. (S14)

$$g^{(2)}_{n_e,N}(0,0)=\frac{(n_e-1)(N-n_e+2)}{n_e(N-n_e+1)}\,, \tag{S21}$$

what shows that the atomic system emits nonclassical light with photon number fluctuations smaller than those for coherent light under the condition that $2(n_e-1)<N$.

## Cross correlations in the radiation of $N$ atoms in arbitrary generalized $W$-states

Finally, we study the spatial cross correlations in two-photon superradiance for atoms in the generalized state $|W_{n_e,N}\rangle$, i.e, the behavior of the second order correlation function $g^{(2)}_{n_e,N}(\delta_1,\delta_2)$ in case that the scattered photons are recorded at different

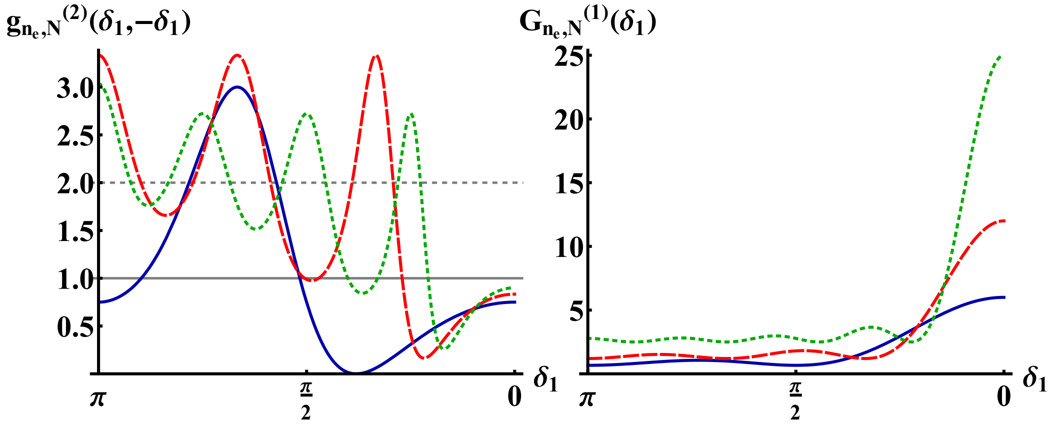


**Figure S2.** Second order correlation function $g^{(2)}_{n_e,N}(\delta_1,-\delta_1)$ (left) and first order correlation function $G^{(1)}_{n_e,N}(\delta_1)$ (right) for $(n_e,N)=(2,3)$ (solid), $(3,6)$ (dashed), $(4,8)$ (dotted). For a given $n_e$, the number of atoms $N$ has been chosen in such a way that superbunching is fulfilled for all positions $\tilde{\delta}_1 = a\frac{2\pi}{N}$, with $a=1,2,\ldots<N/2$ (see Eq. (S24)). Again, antibunching is obtained only for $n_e=2$, independently of $N$ (see Eq. (S22)). For $\delta_1=0$ the second order correlation function displays nonclassicality in case that $2(n_e-1)<N$ (see Eq. (S21)).

positions $\delta_1 \neq \delta_2$. We start to explore the particular configuration of counter-propagating detectors, i.e., detectors at positions $\delta_1 = -\delta_2$, followed by the case where $\delta_2$ is fixed and only $\delta_1$ is varied.

For the case $\delta_1=-\delta_2$, the second order correlation function simplifies to (cf. Eq. (S13))

$$\begin{aligned} g^{(2)}_{n_e,N}(\delta_1,-\delta_1) = \mathcal{N}\binom{N}{n_e}^{-1}\Bigg[&\binom{N-2}{n_e-2}N\left[N-2+N\chi^2(2\delta_1)\right] \\ &-\binom{N-3}{n_e-2}2N\left[(N-4)\left(1-N\chi^2(\delta_1)\right)+N\chi^2(2\delta_1)-N^2\chi^2(\delta_1)\chi(2\delta_1)\right] \\ &+\binom{N-4}{n_e-2}\left[2N(N-3)-4N^2(N-2)\chi^2(\delta_1)+\left(N\chi(2\delta_1)-N^2\chi^2(\delta_1)\right)^2\right]\Bigg]. \end{aligned} \tag{S22}$$

In analogy to the discussion of bunching and superbunching for $\delta_1=\delta_2$ above, we investigate the position $\delta_1=\pi$ (see Fig. S2). Considering again even and odd $N$ separately, we obtain

$$\begin{aligned} g^{(2)}_{n_e,N_{even}}(\pi,-\pi) &= g^{(2)}_{n_e,N_{even}}(\pi,\pi) \\ g^{(2)}_{n_e,N_{odd}}(\pi,-\pi) &= g^{(2)}_{n_e,N_{odd}}(\pi,\pi)\,. \end{aligned} \tag{S23}$$

From these expressions it follows that superbunching occurs equivalently for $\delta_1=\delta_2=\pi$ and $\delta_1=-\delta_2=\pi$.

In case of minimal intensity $\chi(\delta_1)=\chi(2\delta_1)=0$, i.e., at positions $\tilde{\delta}_1 = a\frac{2\pi}{N}$, where $a=1,2,\ldots<N/2$, the following form of the correlation function is obtained

$$g^{(2)}_{n_e,N}(\tilde{\delta}_1,-\tilde{\delta}_1) = \frac{(N-1)(4+N^2+2N-6n_e-2Nn_e+2n_e^2)}{(N-2)(n_e-1)n_e}\,. \tag{S24}$$

In principal, for $N \gg n_e$, there is again no upper limit to the correlation function, since $g^{(2)}_{n_e,N}(\tilde{\delta}_1,-\tilde{\delta}_1) \sim N^2/(n_e(n_e-1))$. This behavior is equivalent to $g^{(2)}_{n_e,N_{even}}(\pi,\pi)$ (cf. Eq. (S16)), but valid also for odd $N$. When setting $n_e=3$, bunching occurs for $N\geq 5$ while superbunching occurs for $N \geq 6$ (cf. Eq. (S17)).

Fig. S2 shows the plotted correlation function $g^{(2)}_{n_e,N}(\delta_1,-\delta_1)$ for three different combinations $(n_e,N)=(2,3),(3,6),(4,8)$. These combinations have been chosen to fulfill the condition for superbunching for minimal number of atoms $N$ at the positions $\tilde{\delta}_1$. Again, it can be observed that the two-photon superradiance decreases with increasing $n_e$, so that higher numbers of $N$ are required to produce correlations comparable to the case $n_e=2$.

Since for more than two excitations $n_e>2$ the visibility of $g^{(2)}_{n_e,N}(\delta_1,\delta_2)$ is smaller than one, true antibunching cannot be fulfilled. However, cross correlations smaller than one and therefore nonclassical light can be found for $\delta_1=-\delta_2=0$ in case that $2(n_e-1)<N$, as has been discussed for $\delta_1=\delta_2$ (see Eq. (S21)).

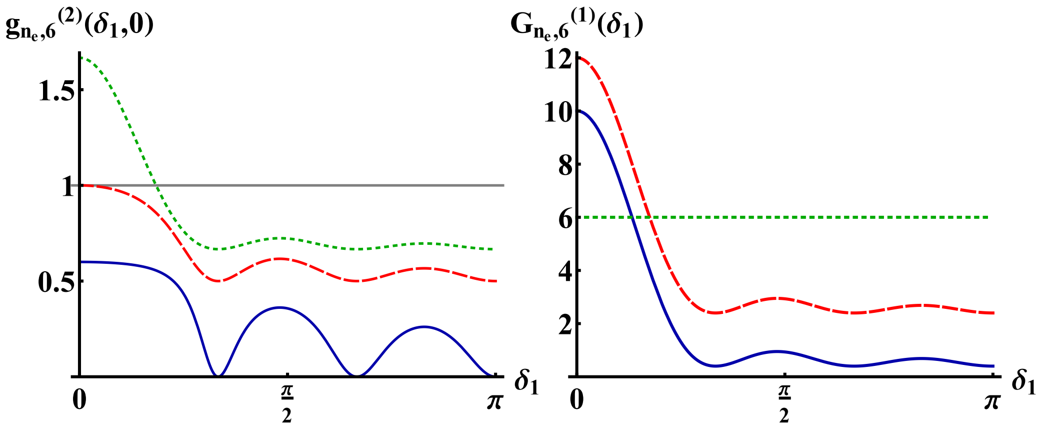


**Figure S3.** Second order correlation function $g^{(2)}_{n_e,N}(\delta_1,0)$ (left) and first order correlation function $G^{(1)}_{n_e,N}(\delta_1)$ (right) for $N=6$ and $n_e=2$ (solid), $n_e=4$ (dashed), $n_e=6$ (dotted). Again, antibunching is observed only for $n_e=2$, independently of $N$, whereas superbunching can never be obtained (see Eq. (S25)).

Another interesting case is the combination of one detector fixed at position $\delta_2=0$ and the second detector moving. Here, the photon cross correlation takes the form (cf. Eq. (S4))

$$g^{(2)}_{n_e,N}(\delta_1,0)=\frac{(n_e-1)(n_e-2)+(n_e-1)(N-n_e+1)N\chi^2(\delta_1)}{n_e(n_e-1)+n_e(N-n_e)N\chi^2(\delta_1)}\ , \tag{S25}$$

which does not acquire values higher than two so that superbunching can never be obtained (see Fig. S3 for $N=6$). The reason is the following: If $g^{(2)}_{n_e,N}(\delta_1,0)<1$ the following condition must be fulfilled

$$(2n_e-N-1)N\chi^2(\delta_1)<2(n_e-1)\ . \tag{S26}$$

In case of $\chi(\delta_1)=0$ the relation Eq. (S26) simplifies to

$$0<2(n_e-1)\ , \tag{S27}$$

which is fulfilled for all $n_e\geq 2$. The case $\chi(\delta_1)=1$ (for $\delta_1=0$) already has been investigated in connection with $g^{(2)}_{n_e,N}(0,0)$ and fulfills $g^{(2)}_{n_e,N}(0,0)<1$ for $2(n_e-1)<N$; for $n_e=N$, $g^{(2)}_{n_e,N}(0,0)$ becomes maximal and reads

$$g^{(2)}_{N,N}(0,0)=2-\frac{2}{N}<2\ , \tag{S28}$$

what proves the above statement.

As concerns antibunching, according to Eq. (S27), nonclassicality occurs for all $n_e\geq 2$ whereas true antibunching does only occur for $n_e=2$, as in all other cases the visibility of $g^{(2)}_{n_e,N}(\delta_1,\delta_2)$ remains smaller than one (cf. Eq. (S25)).

## References


1. Auffèves, A., Gerace, D., Portolan, S., Drezet, A. & Santos, M. F. Few emitters in a cavity: from cooperative emission to individualization. *New Journal of Physics* **13**, 093020 (2011).
2. Wiegner, R., von Zanthier, J. & Agarwal, G. S. Quantum-interference-initiated superradiant and subradiant emission from entangled atoms. *Phys. Rev. A* **84**, 023805 (2011).
